# A single-cell atlas of the developing *Drosophila* ovary identifies follicle stem cell progenitors

**DOI:** 10.1101/gad.330464.119

**Published:** 2020-02-01

**Authors:** Maija Slaidina, Torsten U. Banisch, Selena Gupta, Ruth Lehmann

**Affiliations:** Department of Cell Biology, Howard Hughes Medical Institute, Skirball Institute of Biomolecular Medicine, New York University School of Medicine, New York 10016, USA

**Keywords:** *Drosophila*, gene expression signature, single-cell RNA sequencing, cluster analysis, lineage analysis, ovary development

## Abstract

Addressing the complexity of organogenesis at a system-wide level requires a complete understanding of adult cell types, their origin, and precursor relationships. The *Drosophila* ovary has been a model to study how coordinated stem cell units, germline, and somatic follicle stem cells maintain and renew an organ. However, lack of cell type-specific tools have limited our ability to study the origin of individual cell types and stem cell units. Here, we used a single-cell RNA sequencing approach to uncover all known cell types of the developing ovary, reveal transcriptional signatures, and identify cell type-specific markers for lineage tracing. Our study identifies a novel cell type corresponding to the elusive follicle stem cell precursors and predicts subtypes of known cell types. Altogether, we reveal a previously unanticipated complexity of the developing ovary and provide a comprehensive resource for the systematic analysis of ovary morphogenesis.

## Abstract (precis)

In this study, Slaidina et al. use single-cell RNA sequencing to identify transcription signatures for each cell type in the developing *Drosophila* gonad. Using this cell atlas, the authors identify the long elusive follicle stem cell precursors, uncover subcell types for two somatic cell types and identify driver lines for each of the larval gonadal cell types.

Organs are often maintained by tissue-specific adult stem cells, which reside in specialized niches and contribute to tissue maintenance during the lifetime of the organism. These niche:stem cell compartments are established during development and are tightly regulated in adulthood to ensure organ homeostasis in changing environmental conditions and during aging. Dissecting the origins and molecular mechanisms of adult stem cell specification and morphogenesis is challenging. In many systems, it is unclear whether adult stem cells are direct descendants of embryonic progenitors or whether they are specified later during development.

*Drosophila melanogaster* is a genetically tractable organism and their ovaries have served as a model for adult stem cell studies for decades. However, addressing cell type-specific functions and how cells interact with each other to establish an adult organ has been hampered by lack of cell type-specific tools and markers. Here, we report on a comprehensive single cell atlas of the developing *Drosophila* ovary and identify the progenitors of adult stem cell units. *Drosophila* ovaries house two adult stem cell units—germline stem cell (GSC) and follicle stem cell (FSC) (Dansereau and Lasko 2008)—thus providing an excellent model system to study adult stem cell development and regulation in a genetically tractable organism. The major ovary function, egg production, is achieved by coordinated proliferation and differentiation of GSCs and FSCs, which are both regulated by specialized somatic niche cells. The GSC daughter cells differentiate into eggs, while cells deriving from FSCs give rise to an essential follicle epithelium that covers and nurtures the egg and provides the developing oocyte with essential axial patterning information (Riechmann and Ephrussi 2001). Numerous studies of GSCs have revealed key principles of niche:stem cell signaling, and delivered a wealth of knowledge of GSC development and establishment. However, the exact origin of FSCs remains elusive, their development has yet to be studied, and a clear definition of the stem cell pool is lacking (Nystul and Spradling 2007; Reilein et al. 2017). In addition to GSCs and FSCs, ovaries contain a number of other somatic cell types that support the development and adult functions of the ovary. During development, their proliferation, movement, and differentiation needs to be coordinated to establish a functional adult organ. How this is orchestrated and the exact function of individual cell types remains to be elucidated. This knowledge gap is partly caused by a lack of cell type-specific markers and tools.

Single-cell RNA sequencing (scRNA-seq) allows capture of individual cells of an entire organ to sequence their transcriptomes (Stuart and Satija 2019). We applied this technology to developing fly ovaries to gain a systems view of the complete repertoire of ovarian cell types and their functions during development. For our studies, we chose the late third larval instar (LL3) stage for two reasons. First, specific progenitor populations for the majority of cell types are thought to be established by this stage and, second, germ cells transition from undifferentiated primordial germ cells to self-renewing germline stem cells that reside adjacent to their somatic niches and produce more proximally located differentiating cysts, which will give rise to the eggs (Fig. 1A; Gilboa 2015).

**Figure 1.**
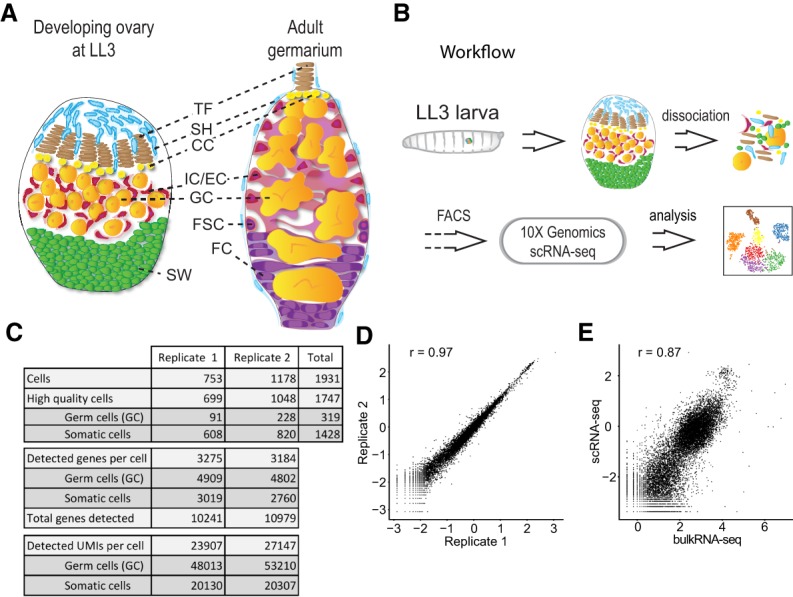
scRNA-seq experiment design and statistics. (*A*) Schematic of a developing ovary, and adult germarium. The drawings are not to scale. (SH) Sheath cells; (TF) terminal filament cells; (CC) cap cells; (IC) intermingled cells; (EC) escort cells; (GC) germ cells; (SW) swarm cells; (FSC) follicle stem cells; (FC) follicle cells. (*B*) scRNA-seq experiment workflow. (*C*) scRNA-seq experiment statistics. (*D*) Gene expression averaged among individual cells in each replicate and compared with each other. (*E*) Gene expression in replicate 1 averaged among individual cells and compared with bulk RNA-seq.

Using scRNA-seq, we identified all known ovarian cell types, additional subtypes, and a novel cell type. By lineage tracing and genetic cell ablation experiments, we demonstrated that this novel cell type corresponds to the long sought after FSC and follicle cell (FC) progenitors. Furthermore, we computed transcriptional signatures for all cell types in the developing ovary, started predicting their function using gene list annotation tools, and selected cell type-specific markers that can be used for further interrogation of cell type function and lineage tracing. Our work provides a resource for future morphogenesis studies of niche:stem cell unit establishment and gonadal support cell function.

## Results

### Single-cell RNA sequencing of developing *Drosophila* ovaries

For single-cell RNA sequencing (scRNA-seq) analysis, we dissected ovaries from developing larvae at LL3 stage that expressed a His2AV::GFP transgene. In these animals, all cell nuclei were labeled with GFP (Supplemental Fig. S1A), allowing cell purification from debris by fluorescence-activated cell sorting (FACS) (Fig. 1B). scRNA-seq was performed on two independently collected samples using the 10× Genomics Chromium system for complementary DNA (cDNA) synthesis and amplification, library preparation, and sequencing. We obtained 753 and 1178 single-cell transcriptomes from ∼15 and 45 larval ovaries, respectively, and used Seurat v2 (Satija et al. 2015; Butler et al. 2018) pipeline to perform established quality control (QC) steps. By plotting the number of genes detected per cell transcriptome, we uncovered two distinct cell populations, separated by the number of genes detected (Supplemental Fig. S1B). Subsequent analyses using known germ cell marker genes (including, *vas*, *AGO3* and others) determined that the population with higher number of genes detected are germ cells (4930 ± 36 in germ cells vs. 2931 ± 17 in somatic cells [mean ± SEM]) (see Fig. 1C; Supplemental Fig. S1C; Supplemental Material). Moreover, we detected a higher number of unique molecular identifiers (UMIs) in germ cells than in somatic cells (53,531 ± 1001 vs. 21,097 ± 27) (Fig. 1C; Supplemental Fig. S1D), suggesting that germ cells contain higher RNA levels than somatic cells. Therefore, we manually separated germ cell transcripts from somatic cell transcripts for initial QC steps (Supplemental Material). Subsequently, we retained 699 and 1048 high-quality cell transcriptomes from the two samples, respectively. Gene expression levels highly correlated between both replicates (Spearman = 0.97) (Fig. 1D) and between our scRNA-seq data set and bulk RNA-seq generated from dissected LL3 ovaries (Spearman = 0.87) (Fig. 1E) despite different library preparation experiments (see the Materials and Methods). Thus, our sample preparation methods are robust and did not significantly alter ovarian transcription profiles. Together, scRNA-seq of dissected developing ovaries yielded a high-quality data set containing 1747 ovarian cell transcriptomes.

### The cell types of the developing ovary

**Figure 2.**
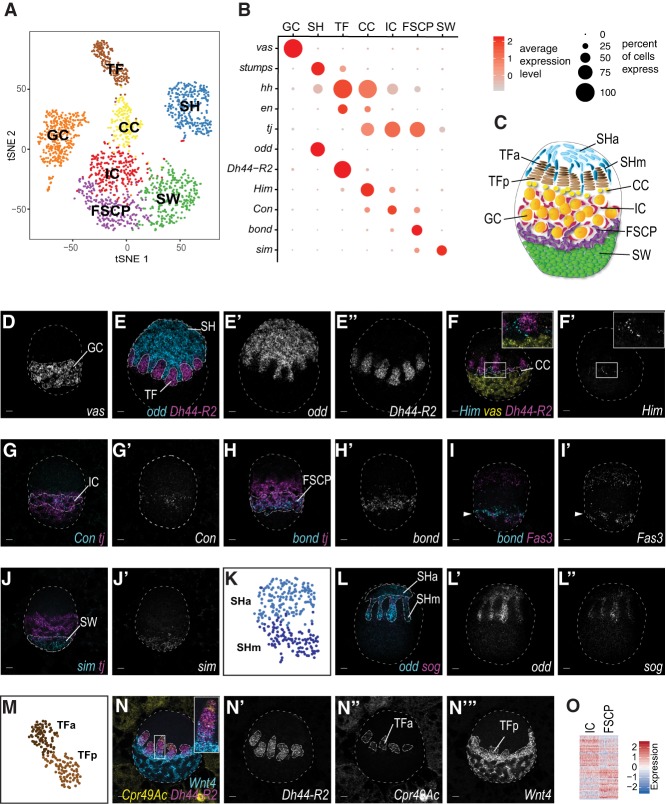
scRNA-seq reveals 7 cell types in developing *Drosophila* ovaries. (*A*) Visualization of cell clusters using t-distributed stochastic neighbor embedding (tSNE). Each point corresponds to a single cell and is color-coded according to cluster membership. (*B*) Visualization of previously described and newly identified marker gene expression in a dot plot. For each gene in each cluster expression levels are indicated by a color gradient; the percentage of cells expressing the gene is indicated by the size of the dot. (*C*) Schematic drawing of a LL3 ovary with newly identified cell types and subtypes. The drawing is not to scale. (*D*–*J*,*L*,*N*) mRNA in situ hybridization using HCR. Scale bars, 10 μm. (*D*) *vas* (grayscale) labels GCs. (*E*) *odd* (cyan, grayscale) labels SH, and *Dh44-R2* (magenta, grayscale) labels TFs. (*F*) *Him* (cyan, grayscale) labels CCs, *Dh44-R2* (magenta) labels TFs, and *vas* (yellow) labels GCs. (*G*) *Con* (cyan, grayscale) labels ICs, *tj* (magenta) labels ICs, and CCs and FSCPs. (*H*) *bond* (cyan, grayscale) labels FSCPs, and *tj* (magenta) labels ICs, CCs, and FSCPs. (*I*) *bond* (cyan) labels FSCPs, and *Fas3* (magenta, grayscale) labels SH, TF, and FSCPs (arrowhead). (*J*) *sim* (cyan, grayscale) labels SWs, and *tj* (magenta) labels ICs, CCs, and FSCPs. (*K*) tSNE plot revealing two SH subclusters. (*L*) *odd* (cyan, grayscale) labels all SH, and *sog* (magenta, grayscale) labels SHm. (*M*) tSNE plot revealing two TF subclusters. (*N*) *Dh44-R2* (magenta, grayscale) labels all TFs, *Cpr49Ac* (yellow, grayscale) labels TFa, and *Wnt4* (cyan, grayscale) labels TFp, CCs, ICs, FSCPs, and SWs. Scale bars, 10 μm. (*O*) Visualization of gene expression levels of genes differentially expressed between IC and FSCP in a heat map. (Red) High expression; (blue) low expression.

Next, we determined the cell type identity for each high-quality cell transcriptome. We batch corrected (aligned) the data sets from the two independent experiments, reduced dimensionality and binned cells into clusters using unsupervised hierarchical clustering (Fig. 2A; Satija et al. 2015; Butler et al. 2018). With multiple clustering parameters, we robustly identified seven clusters (Fig. 2A; Supplemental Fig. S2A). Previous studies had identified six cell types in the larval ovary based on morphology, position, and select gene expression (Fig. 1A): germ cells (GCs) located in the middle of the ovary and five somatic cell populations surrounding the germ cells. Sheath cells (SH) are located at the anterior tip of the LL3 ovary (King et al. 1968). During metamorphosis they will subdivide each ovary into 16–20 units, called ovarioles (King et al. 1968; Irizarry and Stathopoulos 2015). Terminal filaments (TFs) and cap cells (CCs) together form the niche for GSCs (Sahut-Barnola et al. 1996; Song et al. 2007; Gilboa 2015) and are positioned between the SH and GCs. Intermingled cells (ICs) have acquired their name because they intermingle with germ cells and regulate their proliferation (Li et al. 2003; Gilboa and Lehmann 2006). Finally, swarm cells (SW) (also called basal cells) are located at the posterior tip of the LL3 ovary and their function is not known (Couderc et al. 2002; Gilboa 2015).

To correlate the clusters obtained through scRNA-seq with previously described cell types, we identified markers for each cluster based on (1) enrichment of the cluster relative to other clusters, (2) robust level of expression, and (3) expression in a large fraction of cells within a given cluster (Supplemental Table S1). We then compared these markers with previously described cell type marker genes (Fig. 2B; Supplemental Fig. S2C). A germ cell cluster was easily identified by known germ cell-specific genes, such as *vas* (Fig. 2B,D; Supplemental Fig. S2G). In contrast, assignment of somatic cell clusters to specific cell types was more difficult as many genes are expressed in several cell clusters. Nonetheless, we were able to assign SH fate based on *stumps* and *ths* expression (Irizarry and Stathopoulos 2015), TF and CC fates on the basis of high *hh* expression in both (Lai et al. 2017), and alternate enrichment for *en* in TFs (Forbes et al. 1996) and *tj* in CC (Fig. 2B; Supplemental Fig. S2H–K; Li et al. 2003). We confirmed these cell type assignments by assessing the expression patterns of newly identified cluster-specific markers using a highly sensitive method for in situ visualization of RNA: hybridization chain reaction (HCR) (Choi et al. 2018). This analysis revealed that *odd* labels SH (Fig. 2E; Supplemental Fig. S2L), *Dh44-R2* labels TFs (Fig. 2E,F; Supplemental Fig. S2M), and *Him* labels a narrow band of CCs flanked by TF and IC cells, respectively (Fig. 2F; Supplemental Fig. S2N), thus confirming our initial cell type assignments.

To assign identities to the other clusters, we identified cluster-specific markers and determined their expression pattern in the larval ovary (Fig. 2B; Supplemental Table S1). Two clusters expressed *tj*, the transcript for the large MAF transcription factor Traffic Jam. During early larval stages *tj* is expressed in all somatic cells of the ovary and later at LL3 restricts to CCs and a broad band of cells that is closely associated (intermingled) with the medially located germ cells. In the adult, *tj* is expressed in escort cells (ECs) and adult FSCs/FC (Li et al. 2003; Gilboa and Lehmann 2006). We therefore reasoned that the two clusters correspond to the intermingled cells (IC), which would give rise to adult ECs, and the elusive FSC progenitors, which would give rise to follicle stem cells and their progeny in the adult. Two markers, *Con* and *bond* (Fig. 2B,G,H; Supplemental Fig. S2O,P), were differentially expressed between these two clusters. Based on their anatomic position in the larval ovary and presumed fate in the adult, we assigned *Con*-expressing cells to the more anterior located IC population (Fig. 2G) and the *bond*-expressing cells, residing posterior to ICs, to a putative FSC and FC progenitor population (FSCP) (Fig. 2H). In support of this assignment, we observed that the putative FSCPs but not the ICs expressed *Fas3* (Fig. 2I; Supplemental Fig. S2C,Q), which labels follicle cells in adults (Nystul and Spradling 2007).

ICs and the putative FSCP population share a large fraction (∼65%) of their marker genes, yet 54 genes are differentially expressed between the two cell types, supporting the conclusions that these are two distinct cell types within the larval ovary (Fig. 2O). Among the FSCP markers that were not expressed in ICs, we identified CG43693. CG43693's expression overlapped with *bond*, was absent in ICs, but partially also overlapped with *sim* expression (Supplemental Fig. S2R,Z). *sim* was specifically expressed at the posterior tip of the ovary identifying SWs (Fig. 2B,J; Supplemental Fig. S2S).

Taken together, by correlating known and newly identified markers with expression patterns in the developing ovary, we were able to assign seven clusters to distinct ovarian cell types. We obtained gene expression profiles (Supplemental Table S2) of all previously described cell types in developing ovaries and identified a putative progenitor population for the adult FSCs and FCs that nestles between the more anteriorly located ICs and the more posterior SWs. Despite its comprehensive nature, our analysis was limited by both, the number of cells analyzed and the number of markers available for cell type assignment. Thus, we cannot rule out that we missed additional, extremely rare cell types.

### Transcriptionally distinct subtypes divide TF and SH cells

After assigning each cluster with a cell type identity, we searched for systematic transcriptome variability within clusters. For this, we raised the resolution parameters for in silico cell clustering, and as result the GC, TF, and SH clusters split into subclusters (arrowheads in Fig. 2K,M; Supplemental Fig. S2A; Butler et al. 2018). Further investigation suggested that the GC cluster split is unlikely to have biological significance as it was not observed when the cluster was analyzed separate from the somatic cell types (Supplemental Fig. S2F; for further discussion, see the Supplemental Material).

To test the robustness of TF and SH subclusters, we reclustered each cell type independently of other cell types (Supplemental Fig. S2F′,F′′). The gene expression patterns of the independently reclustered SH and TF subclusters clearly corresponded to the initially identified clusters (Spearman = 0.99). Thus, SH and TF subclusters may represent specific subpopulations among SH and TF cells. To determine whether these subpopulations reflect a developmental or morphological distinction within the known cell type, we identified markers that distinguished the subclusters (Supplemental Table S1) and assessed their expression patterns in vivo. For the SH subclusters, we found that SH cells expressing both *sog* and *odd* are migrating between the TF stacks, hereafter referred to as SHm (migrating) (Fig. 2L; Supplemental Fig. S2U,V), and that SH cells, which only express *odd* correspond to the sheath cells located at the anterior tip of the ovary, which we now call SHa (anterior). For TF subtypes, *Dh44-R2* labeled both TF subclusters, while *Cpr4Ac* was expressed only in the anterior half and *Wnt4* labeled the posterior half of the TFs as well as other cell types (CC, IC, FSCPs, and SW) (Fig. 2N; Supplemental Fig. S2W–Y). We refer to these subtypes as TFa and TFp for anterior and posterior, respectively. Thus, individual cell type clustering revealed subpopulations in previously described cell types. It will be of interest to probe their developmental trajectories and biological roles in the future.

### Cell type-specific transcriptional signatures reveal functional connections between cell types

**Figure 3.**
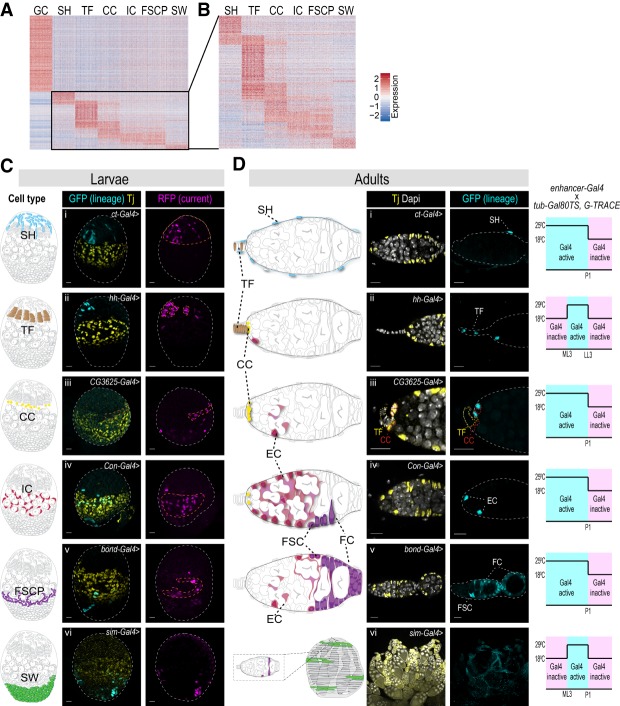
Transcriptional signatures reveal shared functions between cell types. (*A*,*B*) Visualization of cell type signature gene expression in all cell types in a heat map. (Red) High expression; (blue) low expression. (*A*) Transcriptional signature for each cell type. (*B*) Transcriptional signatures of somatic cell types. (*C*,*D*) Immunofluorescence of lineage tracing in larvae (*C*) and adults (*D*) using G-TRACE. (*C*) Schematic drawings in the *left* column indicate the larval cell type that is being targeted: SH using *ct-Gal4* (panel *i*), TF using *hh-Gal4* (panel *ii*), CC using *CG3625-Gal4* (panel *iii*), IC using *Con-Gal4* (panel *iv*), FSCP using *bond-Gal4* (panel *v*), and SW using *sim-Gal4* (panel *vi*). GFP labels the lineage expression (cyan), RFP labels current expression (magenta), and Tj labels ICs, CCs, and FSCPs (yellow). (*D*) Schematic drawings of adult ovarioles indicate cell types where lineage expression is detected. DAPI labels all cell nuclei (white), Tj labels CCs, ECs, FSCs, and FCs (yellow), and GFP labels lineage expression (cyan). (*Right* column) Schematic representation (not to scale) of temperature shifts used to restrict Gal4 activity to larval and/or pupal stages of development to ensure faithful developmental lineage labeling. No RFP (current expression) was observed in the adult ovaries.

Cell states and functions should be reflected by the gene repertoire they express. Thus, the transcriptomes for each cell type in developing ovaries should allow us to explore their respective functions. To enrich for transcriptional signatures that are cell type-specific, we excluded those genes that are uniformly expressed in all cell types and mostly encode proteins associated with general cellular processes (Supplemental Table S1). We also excluded marker genes that were assigned to more than three cell types. We visualized the gene expression levels of the transcriptional signature in each cell type by heat map (Fig. 3A,B; Supplemental Table S3). We then used the gene list annotations for *Drosophila* (GLAD) online resource (Hu et al. 2015), hypergeometric tests, and manual curation to correlate transcriptional signatures with potential functional specializations for each cell type (Supplemental Fig. S3A; Supplemental Table S3).

Germ cells are specified earlier and independently of the somatic gonadal cells. Consistently, germ cells have the highest number of signature genes (1073 genes) (Fig. 3A). The germ cell transcriptional signature was enriched for multiple GLAD categories that fit well with known and prominent features of the germline, including posttranscriptional regulation (splicing, RNA regulation, translation, protein degradation) and mitochondrial maturation and selection (oxidative phosphorylation, autophagy) (Cox and Spradling 2003; Kai et al. 2005; Slaidina and Lehmann 2014; Teixeira et al. 2015; Lieber et al. 2019).

All somatic cells of the gonad arise from a somatic gonadal precursor population that is specified during embryogenesis (Boyle and DiNardo 1995; Moore et al. 1998; Riechmann et al. 1998). The signature gene heat map reflects this shared origin as most cell types can be distinguished from each other by only a small number of genes. We arranged the somatic gonadal cell types along the ovarian anterior-to-posterior axis in a pattern that may reveal developmental relationships between cell types and distinct functional specialization (Fig. 3B). For example, the two most anterior cell types, SH and TFs have clear transcriptional signatures that set them apart from each other and all other somatic gonadal cell types (SH: 179 genes; TF: 404 genes), suggesting that these cell types may have diverged from a common precursor pool early in development (Godt and Laski 1995). Consistent with their common function in forming the germline stem cell niche, CCs and TFs share a fraction of their transcriptional signature (CC: 262 genes; common: 122 genes). Finally, the strongly correlated transcriptional signature of ICs and FSCPs (IC: 155 genes; FSCP: 161 genes; common: 101 genes) suggests that they originated from a common progenitor subpopulation late in development or that they fulfill related functions.

Consistent with their role in germ cell support and gonad morphogenesis, all somatic cell types are enriched for gene ontology terms associated with cell signaling. For example, Notch ligands are enriched in SH and TFs, and all other cell types highly express Notch receptors and/or their downstream pathway components (Song et al. 2007). Multiple somatic cell types are significantly enriched for genes expressing proteins with roles in mediating cell–cell communication. These protein classes will expand previous observations which showed that, in addition to conventional signaling pathways, ovarian cells coordinate their behaviors through alternative modes of signaling (Gilboa et al. 2003; Banisch et al. 2017). For example, the gap junction protein *zpg* is highly enriched in germ cells, while other gap junction proteins, *inx2*, *inx3*, and *ogre,* are expressed in somatic cell types surrounding the germ cells. Cell type-specific enrichment for transcription factors and DNA-binding proteins can provide a useful tool to study gene regulatory networks for gonad development at a cell type resolution. For example, the *bab1* and *bab2* transcription factors are required for the development of TFs and CCs, the GSC niche (Godt and Laski 1995). Adult FSCs are regulated by the Hedgehog signaling pathway (Zhang and Kalderon 2001), and its downstream transcription factor Ci is specifically enriched in FSCPs, suggesting that similar regulation might take place during development as well. For a more detailed analysis and summary of gene classes enriched in specific cell types, refer to Supplemental Figure S3A; Supplemental Table S3.

### Connecting precursors to adult cell types by lineage tracing

**Figure 4.**
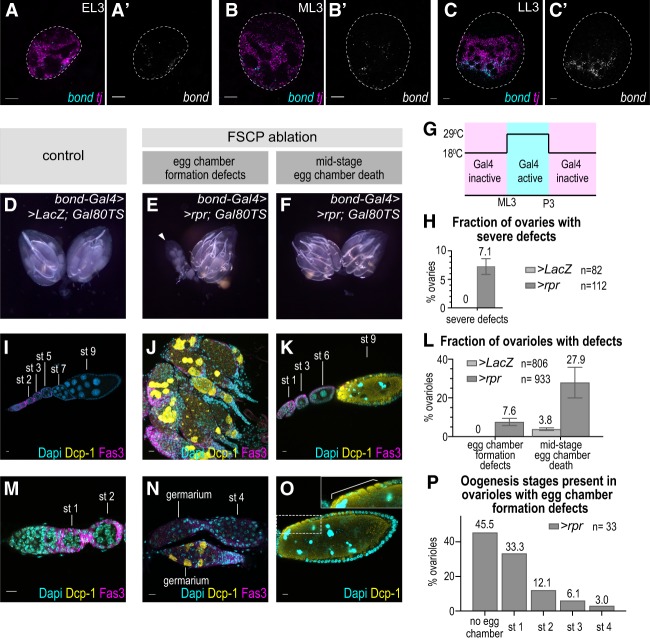
FSCP ablation disrupts normal development of adult FSCs and FCs. (*A*–*C*) mRNA in situ hybridization using HCR at EL3 (*A*), ML3 (*B*), and LL3 (*C*). (Magenta) *tj* ; (cyan, grayscale) *bond*. Scale bars, 10 μm. (*D–P*) Ablation of FSCPs by *bond-Gal4* driven expression of the proapoptotic factor *rpr* restricted to late larval and early pupal stages causes FC defects in adults. (*D*,*I*,*M*) Control. (*E*,*F*,*J*,*K*,*N*,*O*) FSCP ablation by *rpr* expression using *bond-Gal4; Gal80TS*. (*D*–*F*) Wide-field image of entire ovary pair. (*G*) Gal4 activation (29°C) and inactivation (18°C) by Gal80TS is indicated in a schematic drawing. (*H*) Quantification of a number of severely distorted ovaries (shown in *E*). (*I*–*K*,*M*–*O*) Immunofluorescence staining of cleaved Dcp-1 (yellow) labeling apoptotic cells, Fas3 (magenta) labeling follicle cells, and DAPI (cyan) labeling nuclei. Scale bars, 10 μm. (*I*) Control ovariole with a string of egg chambers of various stages indicated *above* each egg chamber. (*J*) Multiple FSCP-ablated germaria with dying germ cells and somatic cells (labeled by Dcp-1). Mature egg chambers are absent from these ovarioles. (*K*) FSCP-ablated ovariole with a string of egg chambers with the most posterior egg chamber dying (labeled by Dcp-1). (*L*) Quantification of frequency of egg chamber formation defects (shown in *J* and *N*) and mid-stage egg chamber death (shown in *K* and *O*) in control and FSCP-ablated ovaries. (*M*) Control germarium with stage 1 and a stage 2 egg chambers. (*N*) FSCP-ablated germarium with severe defects (as in *J*). (*O*) Dying egg chamber from FSCP ablated ovary. A gap in the follicular layer is indicated by a bracket. (*P*) Quantification of oogenesis stages present in ovarioles with egg chamber formation defects.

During metamorphosis, developing ovaries turn into their respective adult structures. However, for only a fraction of adult descendants are the progenitor cell types known. Therefore, we took advantage of our newly identified cell type markers and designed lineage tracing experiments that determined the lineage relationships between defined cell types in developing ovaries and adult descendants. For lineage analysis, we used G-TRACE, a method that in our experimental design combines a cell type-specific Gal4 driver with the UAS-FLP recombinase-FRT systems to generate clones marked with nuclear GFP for lineage analysis and a UAS fluorescent reporter to observe real-time expression patterns (Evans et al. 2009). To identify appropriate driver lines for each larval cell type, we tested 79 publicly available lines with Gal4 integrated near the regulatory sequences of individual somatic cell type marker genes. We first tested the expression pattern of each line at LL3 by costaining with anti-Tj antibodies and Dapi (Fig. 3C). We identified at least one Gal4 driver line for each somatic cell type (Fig. 3C,D; Supplemental Fig. S3B,C). Real-time labeling with the G-TRACE cassette confirmed driver expression predominantly in the predicted cell types (Fig. 3C). In contrast to RNA expression analysis by mRNA in situ hybridization, the drivers showed relatively sparse expression within the tissue of interest and also labeled a few cells most likely associated with other cell types. We used the thermosensitive Gal80 (Gal80TS) repressor to restrict the time interval, at which the drivers were active, to larval or up to early pupal stages only (Fig. 3D). Due to the sparse labeling, we did not expect that all cells of a particular cell type in the adult would be labeled. Therefore, we counted the number of ovarioles with labeling for each cell type (Supplemental Fig. S3C). Dependent on the driver line and cell type analyzed, GFP-labeled cells were detected in between 14% to 54% of adult ovarioles (Supplemental Fig. S3C). Altogether, we were able to follow the cell lineages and determine the cell type lineage relationships between larval and adult ovaries for each cell type identified by RNA sequencing (Fig. 3C,D; Supplemental Fig. S3B,C). For example, *cut*-*Gal4*-labeled SH cells at LL3 gave rise predominantly to the epithelial sheath that surrounds each ovariole in the adult (Fig. 3C [panel i],D [panel i], Supplemental Figs. S3C, S4A,C; Irizarry and Stathopoulos 2015). As described previously, *hh-Gal4* marked TFs continuously from the larva to the adult (Fig. 3C [panel ii],D [panel ii]; Supplemental Fig. S3C; Lai et al. 2017). A *CG3625-Gal4* line predominantly labeled CCs at LL3 (Fig. 3C [panel iii],D [panel iii]; Supplemental Figs. S3C, S4B) and their progeny gave rise to adult CCs and rarely ECs, supporting the notion of common ancestry of these two cell types (Song et al. 2007). Consistently, ICs labeled by *Con*-*Gal4* gave rise to ECs and less frequently to CCs in adults (Fig. 3C [panel iv],D [panel iv]; Supplemental Fig. S3C). The adult descendants of SWs had not been determined. A *sim*-*Gal4* driver labeled SWs at LL3 (Fig. 3C, panel vi); however, we did not detect any robust lineage expression in the adult germarium (besides rare labeling of single SH, EC, and FCs). Instead, we identified lineage-labeled cells in the outer ovarian sheath (also called peritoneal sheath) (Spradling 1993), suggesting that it originates, at least in part, from the larval SW population (Fig. 3D, panel vi; Supplemental Fig. S3C). To follow the putative FSCP population we chose *bond-Gal4*. We found that cells expressing this marker in the larva gave rise predominantly to follicle cells and FSCs in the adult (Fig. 3C [panel v],D [panel v]; Supplemental Fig. S4D). We noted that *bond-Gal4* drives expression in a slightly broader pattern than what was observed with HCR for the *bond* gene in the LL3 ovary (Figs. 2H, 4C); this may explain sparse lineage expression in ECs and some other cell types in the adult (Fig. 3D, panel v). Together, these results suggest that follicle cells are derived from a larval precursor population nested between the ICs, the precursors of the adult ECs, and the SWs.

The Gal4 driver lines we identified for each somatic cell type allowed us to establish lineage trajectories to their adult descendants. As mentioned above, expression of the Gal4 drivers was prominent but not completely confined to a single cell type. This either suggests that the drivers do not fully recapitulate the expression pattern of the targeted gene, or it reflects that some somatic cell types of the LL3 ovary have recently diverged from a common progenitor pool. In this scenario, cell type boundaries are still fluid and the activity of the Gal4 driver line reflects the dynamics of evolving gene expression patterns. We reasoned that the latter might be the case, in particular for the CC, IC, and FSCP lineages. It has been proposed that ICs give rise to CCs, and that adult FC and EC fates might still be in flux at adult stages (Song et al. 2007; Reilein et al. 2017).

### FSCP ablation during development causes follicle cell defects in adults

Expression signatures and lineage labeling identified a putative FSCP population at the LL3 stage. By observing the expression of *bond* RNA during earlier stages of development, we asked when these cells are first specified. We detected *bond* expression first at the early third larval instar stage (EL3, 72 h AEL [after egg laying]) (Fig. 4A; Supplemental Fig. S4E). At this stage, sparse and weak *bond* expression covers the entire *tj* expression domain, suggesting that the FSCPs share common ancestry with ICs, which also express *tj*. At ML3 (96 h AEL) (Fig. 4B; Supplemental Fig. S4F), *bond* expression restricted toward posterior, and a strong band of *bond* expression was present at the posterior part of the *tj* expression domain at LL3, now likely restricted to the FSCP lineage (Fig. 4C; Supplemental Fig. S4G).

It remained unclear whether the putative FSCP population contained only the precursors for the adult FSC or also those for other follicle cell precursor populations. To test which follicle cell populations arise directly from the larval cell population, we ablated the FSCPs between ML3 to the mid-pupal stages using *bond-Gal4* and *Gal80TS-*mediated temporal expression of *reaper* (*rpr*), an apoptosis-inducing gene (Fig. 4G; White et al. 1996). Control adult females developed normal ovaries (Fig. 4D,H), while ovary morphology was grossly abnormal in 7% of FSCP-ablated females (Fig. 4E,F,H). Detailed analyses of FSCP-ablated ovaries revealed defects in 35.5% of all ovarioles. We detected two distinct phenotypes. The first phenotype, which we refer to as “egg chamber formation defects,” was very severe and led to complete egg chamber formation defects, which were generally associated with highly abnormal, distorted ovaries (7.6% of total ovarioles of FSCP-ablated females vs. 0% in controls) (Fig. 4I,J,L). In the majority of ovarioles with this phenotype, oogenesis was blocked before stage 2 (Fig. 4M,N,P), and germline and somatic cells were undergoing apoptosis, as determined by staining for the cleaved *Drosophila* cell death protease Dcp-1 (Fig. 4J,N). The second phenotype, which we refer to as “egg chamber death” was characterized by mid-stage egg chamber death (stages 8–9) and was observed in 27.9% of total ovarioles of FSCP ablated females (compared with 3.8% in controls) (Fig. 4K,L,O). This phenotype was characterized by Dcp-1 staining and pyknotic nurse cell nuclei, hallmarks of egg chamber apoptosis (Pritchett et al. 2009). In these dying egg chambers, we observed gaps in the follicular epithelium revealed by Dlg, a lateral membrane marker of the follicular epithelium (brackets in Fig. 4O; Supplemental Fig. S4H,I; Bilder et al. 2000).

We reasoned that these two distinct phenotypes could arise from the targeted deletion of two FSCP subpopulations that would give rise, respectively, to two adult follicle cell populations: the follicle stem cells (FSC) and prefollicle cell (pre-FC) precursor populations. In adult ovaries, FSCs divide rarely to produce a transient amplifying population called the prefollicle cells, which rapidly proliferate to produce the large pool of follicle cells that is needed to ensheath each egg chamber. These two adult populations express different levels of the adhesion protein Fasciclin 3 (Fas3), which is strong in adult prefollicle cells and weak or absent in FSCs (Nystul and Spradling 2007; Reilein et al. 2017). Analyzing Fas3 levels in the developing ovary, we also observed a low to high anterior to posterior *Fas3* expression gradient within the *bond*-expressing domain at LL3 (Fig. 2I). These results are consistent with the larval ovary possessing both, weak Fas3-expressing precursors for the adult FSC population situated more anterior at the interface to the future escort cells and strong *Fas3* expressers that mark the more posterior located precursors to the pre-FCs. We propose that by ablation of FSC precursors follicle development would be completely abolished, thereby causing the abnormal and grossly distorted ovaries with egg chamber formation defects. Ablation of pre-FCs, on the other hand, would cause follicle epithelial defects, such defective egg chambers would be eliminated during mid-oogenesis due to checkpoint activation as reported previously (Chao and Nagoshi 1999; Pritchett et al. 2009).

## Discussion

The development of *Drosophila* ovaries has been studied for decades. Nevertheless, functional studies of most ovarian cell types have been hindered by a lack of cell type-specific markers and driver lines. Our study has identified cell type-specific marker genes, which now open the targeted use of hundreds of publicly available GFP fusion constructs (Sarov et al. 2016) that can be used for cell labeling, live imaging, and functional studies. For example, a GFP fusion of a highly specific SH marker *drm*, can be used to label SH (Supplemental Fig. S4J–L), and additional lines exist for other cell types. We used a number of publicly available Gal4 drivers for lineage analyses. While some of these were expressed broader than expected from mRNA expression patterns, we were able to identify Gal4 driver lines for lineage tracing of each larval ovary cell type. In particular, these lines helped us to determine the adult descendants of the swarm cells and identified the long sought-after follicle stem cell progenitors. Going forward, the cell type-specific markers identified in this study can be used for further tool building to more specifically and completely target individual cell types (Supplemental Fig. S4L). For example, strategies involving Gal80TS and split-Gal4 systems may improve driver specificity and avoid expression in other tissues (Pfeiffer et al. 2010). Our GLAD analysis grouped cell type signature genes according to their molecular and cellular functions (Hu et al. 2015). Predicted cellular functions and protein classes enriched in each cell type will provide new insight into how cells in the developing ovary interact, how stem cell units are established, and how these precursor cell interactions support the morphogenesis and homeostasis of the adult ovary.

A major finding of our study is the identification of a follicle stem cell and follicle cell progenitor population. Our results show that the transcriptional signatures of FSCPs and ICs are similar. This could indicate that these two cell types are specified from a common progenitor. In support, the FSCP marker gene *bond* is detected as early as EL3 in a broad expression domain spanning both the FSCP and IC progenitors. *bond* may be expressed in the common progenitor pool and later become restricted to the FSCPs, or the *bond*-expressing FSCPs may be initially dispersed and later migrate posteriorly. In addition to common developmental origins, an overlap in transcriptional signatures may also reflect shared functions. Consistently, ICs and FCs both intimately interact with germ cells and guide their differentiation (Wu et al. 2008; Xie 2013; Banisch et al. 2017); thus, analyzing the overlap between the IC and FSCP transcriptional signatures might reveal the nature of IC/FSCP to GC signaling, and shed light on stem cell-to-support cell communication in general.

Altogether, our study provides a systems-wide overview of cell types, and their transcriptional profiles and signatures in the developing *Drosophila* ovary. This resource will facilitate future studies, leading to a better understanding of how stem cell populations are specified, regulated, and maintained in the context of a growing organ, and more general, how a complex interplay of several cell types achieves to build an organ. Future scRNA-seq experiments using additional stages of development (earlier larval, pupal, adult) or using scRNA-seq methods that allow simultaneous lineage tracing, like scGESTALT (Raj et al. 2018) will allow us to identify the complete lineage relationships between the ovarian cell types. Moreover, perturbing functions of individual cell types will provide information about cellular processes that are coordinated between the cells and how this coordination is achieved. Together, our work should provide an invaluable resource for the stem cell and developmental biology research communities.

## Materials and methods

### Experimental model and subject details

#### Fly husbandry

Flies were raised on medium containing yeast, molasses, and cornmeal, and kept at 25°C. The lineage tracing and ablation experiments were performed at 18°C and 29°C as indicated in the text.

### Method details

#### Dissections

For EL3, ML3, and LL3, properly staged larvae were rinsed in PBS (for immunofluorescence) or DPBS (for RNA in situ hybridization) and sexed (if possible). Posterior part of the cuticle was removed using forceps, and specimens were inverted. Intestines were gently removed, leaving the fat body and other organs intact and attached to the cuticle.

For L2, properly staged larvae were rinsed and their anterior was removed, leaving most organs partly extruding from the cuticle.

Female adults were fattened on yeast for 2–3 d. Abdomens were removed using forceps and parts of intestine were removed, leaving ovaries partly covered by abdominal cuticle.

#### Immunofluorescence

All steps were done with gentle rotation. Specimens were fixed in PBS, 0.3% Triton-X (Tx), and 4% paraformaldehyde for 20 min at room temperature with gentle rotation, washed twice with PBS and Tx 1%, and blocked/permeated for 2 h in PBS, 1% Tx, and 5% normal goat serum (NGS) for 2 h at room temperature. Primary antibody was diluted in PBS, 0.3% Tx, 5% NGS, and incubated for 2 h at room temperature or overnight at 4°C. Subsequently, specimens were washed in PBS, 0.3% Tx three times for 20 min at room temperature and in PBS, 0.3% Tx, 5% NGS twice for 30 min. Secondary antibodies and DAPI were diluted in PBS, 0.3% Tx, and 5% NGS and incubated for 2 h at room temperature or overnight at 4°C. Subsequently, specimens were washed in PBS and 0.3% Tx four times for 20 min at room temperature. Finally, specimens were equilibrated in VectaShield mounting medium overnight at 4°C and pieces of larval fat body containing ovaries/adult ovarioles were mounted in VectaShield.

#### RNA in situ hybridization

All steps are done using RNAse free reagents and supplies with gentle rotation, except for steps at 37°C. The protocol was adapted from Choi et al. (2018). Specimens were fixed in PBS, 0.1% Tween (Tw), and 4% paraformaldehyde for 20 min at room temperature; washed twice with PBS and 0.1% Tw at room temperature; and dehydrated with sequential washes with 25%, 50%, 75%, and 100% methanol in PBS for 5 min each on ice. Samples were stored at least overnight (up to 1 wk) at −20°C. Samples were rehydrated with sequential washes with 100%, 75%, 50%, and 25% methanol in PBS on ice; permeated for 2 h in PBS and 1% Tx at room temperature; postfixed in PBS, 0.1% Tw, and 4% paraformaldehyde for 20 min at room temperature; washed twice with PBS and 0.1% Tw for 5 min on ice; washed with 50% PBS and 0.1% Tw/50% 5× SSCT (5× SSC, 0.1% Tween) for 5 min on ice; washed twice with 5× SSCT for 5 min on ice; incubated in probe hybridization buffer for 5 min on ice; prehybridized in probe hybridization buffer for 30 min at 37°C; and hybridized overnight (16–24 h) at 37°C. Probe concentrations were determined empirically, and ranged from 4 to 8 pmol of each probe in 1 mL; probe solution was prepared by adding probes to prewarmed probe hybridization solution. After hybridization, specimens were washed four times with probe wash buffer for 15 min each at 37°C, and twice with 5× SSCT for 5 min each at room temperature. Specimens were equilibrated in amplification buffer for 5 min at room temperature. Hairpin solutions were prepared by heating 30 pmol of each hairpin for 90 sec at 95°C, cooling at room temperature in the dark for 30 min, and subsequently adding the snap-cooled hairpins to 500 μL of amplification buffer at room temperature. Specimens were incubated in hairpin solution overnight (∼16 h) at room temperature, and washed multiple times with 5× SSCT—twice for 5 min, twice for 30 min, and once for 5 min. DAPI was added in the first 30-min wash. Specimens were equilibrated in VectaShield overnight at 4°C and mounted in VectaShield, or further stained using the immunofluorescence protocol (see above).

#### Imaging

Imaging was performed using Zeiss LSM 800 and Zeiss LSM 780 confocal microscopes using 40× oil NA 1.3 objectives.

#### Ovary dissociation

Fifteen to 45 LL3 ovaries were dissected per sample in ice-cold DPBS; the majority of associated fat body was removed with forceps and dissection needles. For dissociation, ovaries were transferred to 9-well glass plates and incubated in dissociation solution (0.5% type I collagenase, 1% Trypsin; 1:250 in DPBS) for 15 min with gentle rotation. The suspension was vigorously pipetted multiple times during the dissociation to enhance the dissociation efficiency. Enzymatic dissociation was stopped by adding Schneider cell culture medium with fetal bovine serum (S-FBS). Starting from this step, all plastic materials—pipet tips, tubes, filters—were coated with S-FBS. Cell suspension was filtered through a custom-made 40-micron cell strainer. The strainer was built by securing nylon mesh in a cap of a 0.2-mL PCR tube and cutting the bottom of the tube and the cap. Upon filtering, dissociated cells were purified by fluorescence-activated cell sorting (FACS) using a 100-µm nozzle on Sony SY3200 cell sorter.

#### 10× Genomics

Chromium single-cell 3′ V2 reagent kits were used for scRNA-seq library preparation following the manufacturer's protocol.

#### Bulk RNA library preparation

RNA was prepared from dissected LL3 ovaries using QuiagenMicro kit. The libraries were prepared with 5 ng of total RNA input using the NuGen Ovation RNA-seq system V2, 7102-32, and the NuGen Ovation ultralow system V2, 0344-32 kits using the manufacturer's protocol. The samples were sequenced in one lane of HiSeq 4000 as paired-end 150.

#### Sequencing

Single-cell RNA-seq analysis was performed for 10× libraries sequenced on paired-end 26/98 Illumina HiSeq 4000 runs.

### Quantification and statistical analysis

#### 10× Genomics data preprocessing

Per-read per-sample FASTQ files were generated using the Illumina bcl2fastq Conversion software (v2.17) to convert BCL base call files outputted by the sequencing instrument into the FASTQ format.

The 10× Genomics analysis software, Cell Ranger (v1.3.1 for replicate 1 and v2.0.0 for replicate 2), specifically the “cellranger count” pipeline, was used to process the FASTQ files in order to align reads to the *Drosophila melanogaster* reference genome (dm6) (Dos Santos et al. 2015) and generate gene-barcode expression matrices. The output of multiple samples from the “cellranger count” pipeline were aggregated using the “cellranger aggr” pipeline of Cell Ranger, normalizing the combined output to the same sequencing depth and recomputing the gene-barcode matrices and expression analysis accordingly for the aggregated data.

#### 10× Genomics data quality control

Seurat 2 package (Butler et al. 2018) was used for all scRNA-seq analysis. In brief, to remove low-quality cells and potential doublets, we filtered out cells in which >5% of reads were from mitochondrial genes, and cells that express <500 genes. We had determined that germ cells express a higher number of genes and UMIs than somatic cells; therefore, to filter out doublets, we set different filtering thresholds for somatic cells and germ cells. We identified germ cells by expression of five highly specific previously known and newly identified germ cell genes: *vas*, *ovo*, *bru1*, *AGO3*, and *CG9926*. We filtered out germ cells in which we detected >90,000 UMIs and somatic cells with >60,000 UMIs.

#### scRNA-seq data analysis

The two scRNA-seq data sets were integrated (aligned) using Seurat v2 (Butler et al. 2018). We followed the Seurat v2 guidelines for identification of variable genes, dimensionality reduction, and cell clustering. We used multiple resolution parameters (1.2–1.7) and obtained similar results (discussed in results). To find markers, we used Wilcox statistical test built in Seurat 2.

#### Transcriptional signatures

To compute transcriptional signatures for GC, SH, TF, CC, IC, FSCP, and SW, we selected all the markers that are assigned to only one, two, or three of these cell types.

#### GLAD analyses

We used the GLAD online tool (Hu et al. 2015) to determine whether the marker genes for each cell type fall into particular gene categories. We used the hypergeometric test to determine whether each gene category is significantly enriched in each cell type's transcriptional signature.

#### Bulk RNA-seq data preprocessing

Per-read per-sample FASTQ files were generated using the Illumina bcl2fastq conversion software (v2.20) to convert per-cycle BCL base-call files outputted by the sequencing instrument into the FASTQ format. The alignment program STAR (v2.4.5a) (Dobin et al. 2013) was used for mapping reads to the *D. melanogaster* reference genome dm6 (Dos Santos et al. 2015) and the application FastQ Screen (v0.5.2) (Wingett and Andrews 2018) was used to check for contaminants. The software featureCounts (Subread package v1.4.6-p3) (Liao et al. 2013, 2014) was used to generate the matrix of read counts for annotated genomic features.

#### scRNA-seq and bulk RNA-seq correlation

The mean expression value was calculated for each gene among all cells in the scRNA seq data sets, transformed to log_10_ scale and plotted against log_10_ scaled counts of bulk RNA-seq data.

### Data and software availability

The scRNA-seq data have been deposited in GEO under accession code GEO GSE131971.

## Supplementary Material

Supplemental Material
