# Supplementary material for: A single-cell atlas of the developing Drosophila ovary identifies follicle stem cell progenitors
Source: Genes Dev. 2020 Feb 1;34(3-4):239–49. doi: 10.1101/gad.330464.119 (PMC7000915; doi:10.1101/gad.330464.119)
Supplement: Supplemental Material [file supp_34_3-4_239__index.html]

A single-cell atlas of the developing Drosophila ovary identifies follicle stem cell progenitors — Supplemental Material 

# A single-cell atlas of the developing *Drosophila* ovary identifies follicle stem cell progenitors

## Supplemental Material

- Supplemental\_Table\_3\_GLAD.csv
- Supplemental\_text.docx
- Supplemental\_FigS1.ai
- Supplemental\_FigS4.ai
- Supplemental\_FigS2.ai
- Supplemental\_Table\_1\_Markers.csv
- Supplemental\_FigS3.ai
- Supplemental\_Table\_2\_AverageExpression.csv
