# Supplementary material for: A single-cell atlas of the developing Drosophila ovary identifies follicle stem cell progenitors
Source: Genes Dev. 2020 Feb 1;34(3-4):239–49. doi: 10.1101/gad.330464.119 (PMC7000915; doi:10.1101/gad.330464.119)
Supplement: Supplemental Material [file supp_gad.330464.119_Supplemental_text.docx]

SUPPLEMENTAL DATA

## Figure S1. scRNA-seq experiment design and statistics.

A – Immunofluorescence staining of developing ovaries at the time of dissections for scRNA-seq (LL3). GFP (cyan) labels all cell nuclei, Vas (yellow) labels GC cytoplasm, and Dapi (magenta) labels DNA. Scale bar - 10 μm. B - Histogram of number of genes detected per cell. C – Box plots depicting number of genes detected in germ cells and somatic cells. D – Box plots of number of UMIs detected in germ cells and somatic cells. Whiskers show min and max value.

## Figure S2. scRNA-seq reveals 7 cell types in developing *Drosophila* ovaries.

A– tSNE plots of cell clusters computed using multiple resolution parameters (indicated on top of each plot). Each dot corresponding to an individual cell transcriptome is color coded according to cluster membership. B – tSNE plot with cell transcriptomes color coded according to the experimental replicate. C – Visualization of previously described marker gene expression in a dot plot. D – Graph displaying fraction of cells in each cell type from the two replicates. E – Table displaying the number of cells of each cell type reported *in vivo* and recovered in our scRNA-seq experiment. F – Re-clustering of GC (F), SH (F’) and TF (F’’) clusters independently of other cell types. Spearman correlation coefficient of average gene expression between the previously (Supplemental Fig. S2A’) and independently obtained clusters are shown in the top panel, high correlation is highlighted in blue. tSNE plots of independently obtained clusters are visualized in the bottom panel. G-Y – Violin plots visualizing gene expression levels (y axis) in each cell cluster (x axis), each dot represents a cell. G - *vas* is equally expressed in both GC clusters. H – *stumps* is highly expressed in SH. I – *en* is predominantly expressed in TFs. J – *hh* is highly expressed in TFs and CCs. K – *tj* is highly expressed in CCs, ICs and FSCPs. L – *odd* is highly expressed in SH. M – *Dh44*-*R2* is specifically expressed in TFs. N – *Him* is specifically expressed in CCs. O – *Con* is predominantly expressed in ICs. P – *bond* is specifically expressed in FSCPs. Q – *Fas3* is expressed in SH, FSCPs and TFs. R – *CG43693* is predominantly expressed in FSCPs and SWs. S – *sim* is specifically expressed in SWs. T – *bam* is equally expressed in both GC clusters. U – *odd* is equally expressed in both SH sub-types. V – *sog* is predominantly expressed in SHm. W – *DH44-R2* is equally expressed in both TF sub-types. X – *Cpr49Ac* is specifically expressed in TFa. Y – *Wnt4* is highly expressed in TFp and in other cell types, but not TFa.

Z – mRNA *in situ* hybridization using HCR. *bond* (cyan, grayscale) labels FSCPs, *CG43693* (magenta, grayscale) labels FSCPs, SWs, and CCs or anterior ICs, *sim* (yellow, grayscale) labels SWs. Scale bar - 10 μm.

## Figure S3. Transcriptional signatures reveal shared functions between cell types.

A – Table of gene annotation enrichment p-values in each cell type. Highly significant p-values are highlighted in purple (p< 0.001). Germ cell enriched gene annotation categories are highlighted in green, signaling related categories in orange, transcription factors in yellow and others in grey. B – table of all driver lines tested using G-TRACE. Drivers with cell type specific expression are highlighted in cyan, with cell type specific but sparse expression in light cyan, and with too broad unspecific expression in magenta. Line IDs indicate stock number in VDRC (v), Bloomington (bl) and Kyoto (DGRC) (ky) stock centers, or reference. C – Table with the lineage tracing result summary. The cell types labeled most frequently are highlighted in cyan, and less frequently in magenta. Note – *bond-Gal4* labels a large number of FCs and only a few ECs in each adult ovariole. Similarly, *sim-Gal4* labels a large number of SW and only a few FSCPs in each LL3 ovary. To determine lineage/current *sim-Gal4* expression in FSCPs, ovaries were co-stained for *bond* mRNA.

## Figure S4. FSCP ablation disrupts normal development of adult FSCs and FCs.

A, B – Violin plots. A – *ct* is specifically expressed in SH. B – *CG3625* has the highest expression in CCs, with lower expression in TFs, ICs and other cell types. Note - while CG3625 mRNA expression is rather broad, the CG3625-Gal4 line v202570, drives expression predominantly in CCs. C, D - Immunofluorescence of lineage tracing using *ct-Gal4* for SH (C), *bond-Gal4* for FSCPs (D), GFP in cyan labels the lineage expression in sheath cells (C), and FSCs and FCs (D). Scale bar - 10 μm. E-G - mRNA *in situ* hybridization using HCR at EL3 (E), ML3 (F), and LL3 (G), *tj* (magenta) and *GFP* probes (cyan) as a negative control for Fig. 4A-C. Scale bar - 10 μm. H, I - Immunofluorescence staining of cleaved Dcp-1 (yellow) labeling apoptotic cells, Dlg (magenta) labeling lateral membranes of the follicular epithelium and Dapi (cyan) labeling nuclei in control (H) and in a dying mid-stage egg chamber from a FSCP ablated ovary (I). Scale bars - 10 μm. J – Violin plot. *drm* is specifically expressed in SH. K – Immunofluorescence staining of *drm-GFP*. GFP (cyan) labels SH, Vas (magenta) labels GCs. Scale bar - 10 μm. L – Violin plots visualizing expression patterns of nine most selectively expressed genes for each cell type.

# Supplemental Note.

## QC for scRNA-seq

In both scRNA-seq experiments, we detected a median of over 20,000 unique molecular identifiers (UMI, the barcodes, which label individual mRNAs) per cell that corresponded to more than 3,000 genes per cell. Among all cells in the ovary we detected 10,241 and 10,979 genes (of a total of about 17 thousand genes in *Drosophila*), respectively, in the two samples analyzed (Fig. 1C). We used previously established quality control methods to exclude damaged cells and cell doublets. Cells with a high fraction of reads derived from genes encoded by the mitochondrial genome, an indicator of damaged cells, were excluded (Butler et al., 2018). A standard method for filtering out potential cell doublets, is to exclude transcriptomes in which significantly more genes than the median are detected. However, when we plotted the cell distribution by number of genes expressed (nGene), we did not observe the expected normal distribution, but instead observed two peaks suggesting the existence of two cell populations that differ in the number of genes expressed (Supplemental Fig. S1B). Further investigation revealed that germ cell-specific genes were enriched in the cell population that expressed more genes and contained more transcripts (Supplemental Fig. S1CD). Therefore, we separated germ cells from somatic cells using a set of previously known and newly identified germ cell specific marker genes, and set different filtering thresholds for the number of expressed genes in the germline and soma populations (see Methods).

## Higher levels of transcripts in germ cells

We detected more transcripts associated with a higher number of genes in germ cells compared to any of the somatic cell types. This finding could indicate that more genes are active in germ cells compared to somatic cells. In support of this hypothesis, a recent study linked the increased number of expressed genes to a transcriptional scanning mechanism that may correct DNA damage during mouse spermatogenesis (Xia et al., 2018). Alternatively, higher RNA levels may indicate a higher RNA content and, since only a small fraction of all mRNAs in the cell is sequenced, this larger sequence pool could give the impression that more distinct genes are active. Consistent with this, we detected a higher number of UMIs (unique molecular identifiers) in germ cells compared to somatic cells (Supplemental Fig. S1D). Indeed, germ cells are known for their reliance on post-transcriptional regulation (Slaidina and Lehmann, 2014) and mRNAs can be stored for days in germ cells before they are translated (Tadros and Lipshitz, 2005). Thus, while we cannot rule out that more genes are active in larval ovarian germ cells, a more stable RNA pool could equally account for the observed increase in transcript level and thereby give the appearance of a higher active gene pool in germ cells compared to somatic cells.

## QC for cluster analysis and cell type identification

After assigning clusters to cell types, we performed additional quality control steps to ensure robustness of our dataset. First, we assessed whether all cell types are recovered in each replicate. Indeed, cells did cluster entirely depending on their cell type identity irrespective of the replicate (Supplemental Fig. S2B). Moreover, we quantified the fraction of cells attributed to each cell type from the two replicates demonstrating that overall ~40% of cells are from replicate 1 and 60% from replicate 2, and that similar proportions are conserved for all cell types (Supplemental Fig. S2D).

Next, we asked whether the number of cells attributed to each cell type by cluster analysis corresponded to previous data derived from cell counts of cell types identified by morphology or expression pattern in *in vivo* preparations. Such cell counts have been determined for GC, TF and *tj*-expressing cells which include CC, IC and FSCP. As shown in Supplemental Fig. S2E, despite expected variability due to genetic backgrounds and between individual ovaries, the cell numbers we recovered by cluster analysis corresponded closely to the numbers described in the literature (Sarikaya and Extavour, 2015).

## 4. Key marker gene functions

| Symbol | Name | Function |
| --- | --- | --- |
| bond | james bond | Very long chain fatty acid elongase |
| CG3625 | CG3625 | Putative membrane component |
| CG43693 | CG43693 | Putative amino acid transporter |
| Con | Connectin | Transmembrane homophilic cell adhesion protein |
| Cpr49Ac | Cuticular protein 49Ac | Insect cuticule protein |
| ct | cut | Transcription factor |
| Dh44-R2 | Diuretic hormone 44 receptor 2 | Diuretic hormone receptor |
| drm | drumstick | Transcription factor |
| Fas3 | Fasciclin 3 | Cell adhesion molecule |
| hh | hedgehog | Hh signaling pathway ligand |
| Him | Holes in muscle | Tanscriptional corepressor gro binding protein |
| odd | odd skipped | Transcription factor |
| sim | single-minded | Transcription factor |
| sog | short gastrulation | Secreted BMP antagonist |
| tj | traffic jam | Transcription factor |
| vas | vasa | DEAD-box RNA helicase, highly conserved germ cell marker. |
| Wnt4 | Wnt oncogene analog 4 | Wnt protein |

## 5. GC sub-clusters

When using higher resolution parameters for cell clustering, the GC cluster split into sub-clusters (Supplemental Fig. S2A, arrowheads). Closer analysis of gene expression patterns in the split germ cell cluster did not support the hypothesis that the two sub-clusters may correspond to distinct germ line subpopulations, for example, GSCs and their differentiating progeny. First, expression of *bam,* which would be expected enriched in the differentiating cell population was the same in both sub-clusters (0.33 vs 0.42 in GCa and GCb, respectively, p-val = 0.11; Supplemental Fig. S2T). Second, the sub-clusters did not present a clear signature. Importantly, when GCs were re-clustered separately from the somatic cell types, this specific sub-clustering was no longer observed (Supplemental Fig. S2F). This is in stark contrast to the sub-cell types that we found among the TF and SH cluster, which were observed similarly in the analysis of the entire ovary and in cluster-specific analysis. We conclude that the GC cluster split is unlikely to have biological significance but rather reflects an analytical error caused by unsupervised clustering of two very different cell populations, germ cells and somatic cells, with strikingly different expression levels and profiles.

# KEY RESOURCES TABLE

| REAGENT or RESOURCE | SOURCE | IDENTIFIER |
| --- | --- | --- |
| Antibodies | | |
| Chicken polyclonal anti GFP | Aves Labs Inc. | # GFP1020 |
| Rat monoclonal anti RFP | Chromotek | # 5F8 |
| Guinea pig polyclonal anti Tj | Dorothea Godt Lab |  |
| Mouse anti Dcp-1 | Cell Signaling Tech. | # 9578 |
| Rabbit anti Vasa | Lehmann lab |  |
| Mouse anti Dlg | DSHB | # 4F3 |
|  |  |  |
| Chemicals, Peptides, and Recombinant Proteins | | |
| DPBS (no calcium, no magnesium) | Thermo Fisher Scientific | Cat#14190144 |
| Trypsin 1:250 | Thermo Fisher Scientific | Cat#27250018 |
| Type I Collagenase | Invitrogen (Thermo Fisher Scientific) | Cat#17018029 |
| Chromium Single Cell 3’ Library & Gel Bead Kit v2 | 10x Genomics | Cat#PN-120237 |
| Chromium Single Cell A Chip Kit |  | Cat#PN-1000009 |
|  |  |  |
| Critical Commercial Assays | | |
| In Situ HCR v3.0 mRNA Imaging Kit (HCR probe sets, HCR amplifiers, HCR buffers (Probe hybridization buffer, Probe wash buffer, Amplification buffer) | Molecular Instruments | Custom |
| Experimental Models: Organisms/Strains | | |
| *Drosophila melanogaster : w^1118^* | Lehmann lab stock |  |
| *Drosophila melanogaster : His2AV::GFP :* [w^1118^](http://flybase.org/reports/FBal0018186.html); [P{His2Av^T:Avic\GFP-S65T^}62A](http://flybase.org/reports/FBti0017565.html) | (Clarkson and Saint, 1999) | BDSC # 5941 |
| *Drosophila melanogaster : drm-GFP : 2XTY1-T2A-SGFP-NLS-3XFLAG* | (Sarov et al., 2016) | VDRC # [318404](https://stockcenter.vdrc.at/control/product/~VIEW_INDEX=0/~VIEW_SIZE=100/~product_id=318404) |
| *Drosophila melanogaster : GTRACE :*  w[*]; P{w[+mC]=UAS-RedStinger}4, P{w[+mC]=UAS-FLP.D}JD1, P{w[+mC]=Ubi-p63E(FRT.STOP)Stinger}9F6/CyO | (Evans et al., 2009) | BDSC # 28280 |
| *Drosophila melanogaster : GTRACE :*  w[*]; P{w[+mC]=UAS-RedStinger}6, P{w[+mC]=UAS-FLP.Exel}3, P{w[+mC]=Ubi-p63E(FRT.STOP)Stinger}15F2 | (Evans et al., 2009) | BDSC # 28281 |
| *Drosophila melanogaster : cut*-Gal4 : VT058382.GAL4@attP2 | (Tirian and Dickson, 2017) | VDRC # 204071 |
| *Drosophila melanogaster : CG3625*-Gal4 : VT000131.GAL4@attP2 | (Tirian and Dickson, 2017) | VDRC # 202570 |
| *Drosophila melanogaster : Con*-Gal4 : VT025803.GAL4@attP2 | (Tirian and Dickson, 2017) | VDRC # 202269 |
| *Drosophila melanogaster : bond-Gal4 :* PBac{IT.GAL4}bond[1385-G4] | (Gohl et al., 2011) | BDSC # 65697 |
| *Drosophila melanogaster : sim-Gal4 :* w[*]; P{w[+mC]=GAL4-sim.3.7}2/CyO; P{w[+mC]=GAL4-sim.3.7}3 | (Shen et al., 2013) | BDSC # 9150 |
| *Drosophila melanogaster : hh*-Gal4 | (Tanimoto et al., 2000) |  |
| *Drosophila melanogaster : UAS-rpr :* w[1118]; P{w[+mC]=UAS-rpr.C}14 | (Aplin and Kaufman, 1997) | BDSC # 5824 |
|  |  |  |
| Software and Algorithms | | |
| Cell Ranger v1.3.1, v2.0.0 | 10x Genomics |  |
| Seurat 2 | (Butler et al., 2018) |  |
| STAR v2.4.5a | (Dobin et al., 2013) |  |
| featureCounts (Subread package v0.5.2) | (Liao et al., 2013; 2014) |  |
| FastQ Screen(c0.5.2) | (Wingett and Andrews, 2018) |  |
| Fiji | (Schindelin et al., 2012) |  |

SUPPLEMENTAL REFERENCES

Aplin, A.C., and Kaufman, T.C. (1997). Homeotic transformation of legs to mouthparts by proboscipedia expression in Drosophila imaginal discs. Mechanisms of Development *62*, 51–60.

Clarkson, M., and Saint, R. (1999). A His2AvDGFP fusion gene complements a lethal His2AvD mutant allele and provides an in vivo marker for Drosophila chromosome behavior. DNA Cell Biol. *18*, 457–462.

Gohl, D.M., Silies, M.A., Gao, X.J., Bhalerao, S., Luongo, F.J., Lin, C.-C., Potter, C.J., and Clandinin, T.R. (2011). A versatile in vivo system for directed dissection of gene expression patterns. Nat Meth *8*, 231–237.

Sarikaya, D.P., and Extavour, C.G. (2015). The Hippo pathway regulates homeostatic growth of stem cell niche precursors in the Drosophila ovary. PLoS Genet *11*, e1004962.

Schindelin, J., Arganda-Carreras, I., Frise, E., Kaynig, V., Longair, M., Pietzsch, T., Preibisch, S., Rueden, C., Saalfeld, S., Schmid, B., et al. (2012). Fiji: an open-source platform for biological-image analysis. Nat Meth *9*, 676–682.

Shen, S.P., Aleksic, J., and Russell, S. (2013). Identifying targets of the Sox domain protein Dichaete in the Drosophila CNS via targeted expression of dominant negative proteins. BMC Dev Biol *13*, 1.

Tadros, W., and Lipshitz, H.D. (2005). Setting the stage for development: mRNA translation and stability during oocyte maturation and egg activation inDrosophila. Dev. Dyn. *232*, 593–608.

Tanimoto, H., Itoh, S., Dijke, ten, P., and Tabata, T. (2000). Hedgehog creates a gradient of DPP activity in Drosophila wing imaginal discs. Molecular Cell *5*, 59–71.

Tirian, L., and Dickson, B.J. (2017). The VT GAL4, LexA, and split-GAL4 driver line collections for targeted expression in the Drosophila nervous system. bioRxiv *118*, 401.

Wingett, S.W., and Andrews, S. (2018). FastQ Screen: A tool for multi-genome mapping and quality control. F1000Res *7*, 1338.

Xia, B., Baron, M., Yan, Y., Wagner, F., Kim, S.Y., Keefe, D.L., Alukal, J.P., Boeke, J.D., and Yanai, I. (2018). Widespread transcriptional scanning in testes modulates gene evolution rates. bioRxiv 282129.
