# Supplementary figures and images for: A single-cell atlas of the developing Drosophila ovary identifies follicle stem cell progenitors
Source: Genes Dev. 2020 Feb 1;34(3-4):239–49. doi: 10.1101/gad.330464.119 (PMC7000915; doi:10.1101/gad.330464.119)

Figure S1

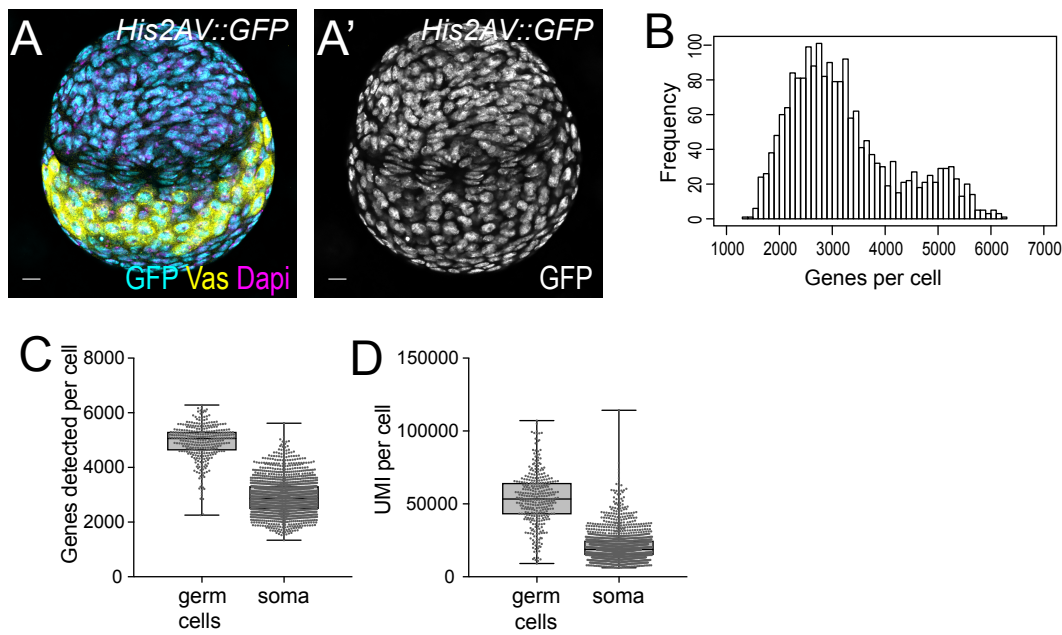

Supplement: Supplemental Material [file supp_gad.330464.119_Supplemental_FigS1.ps]

Figure S4

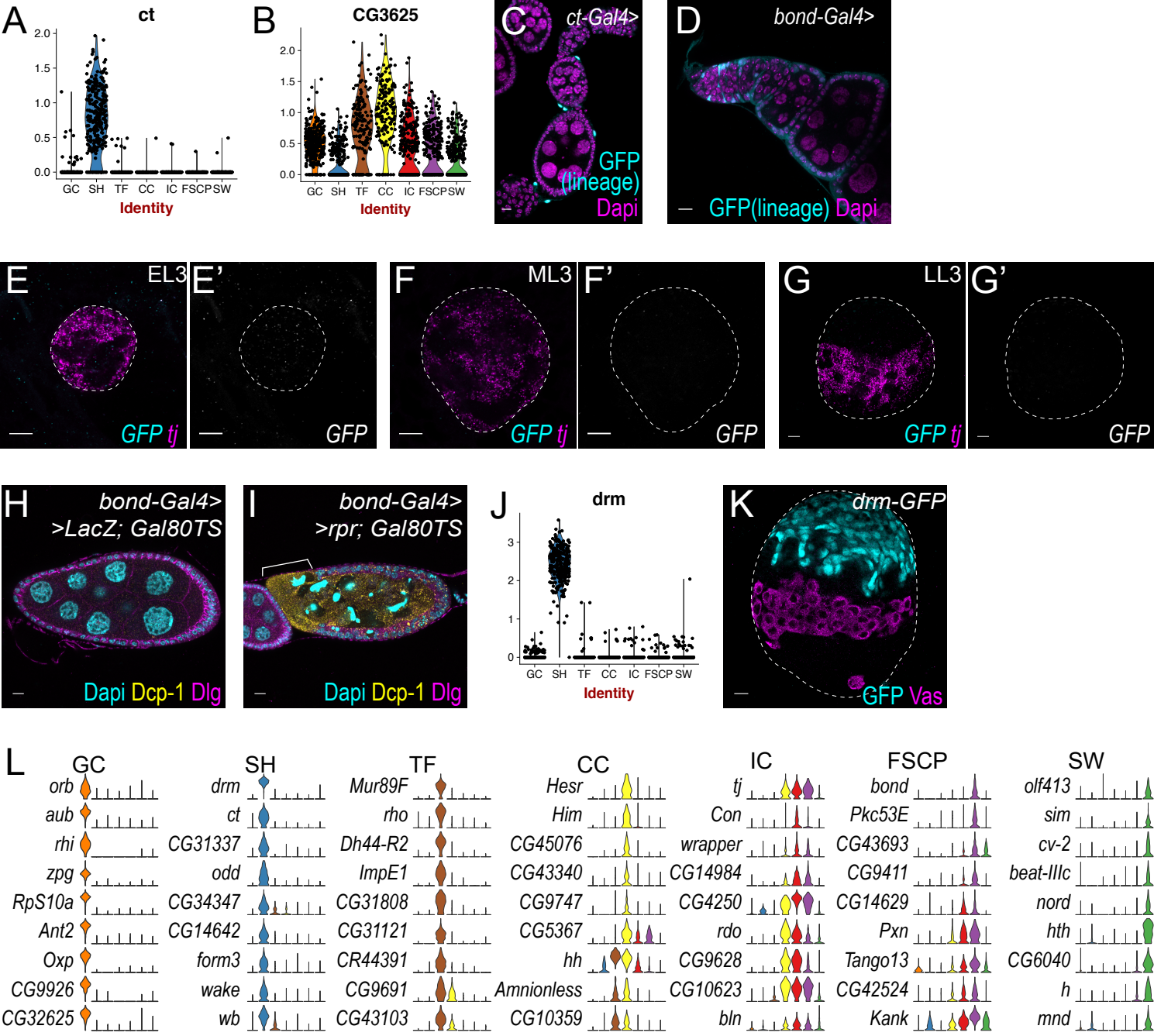

Supplement: Supplemental Material [file supp_gad.330464.119_Supplemental_FigS4.ps]

# Figure S2

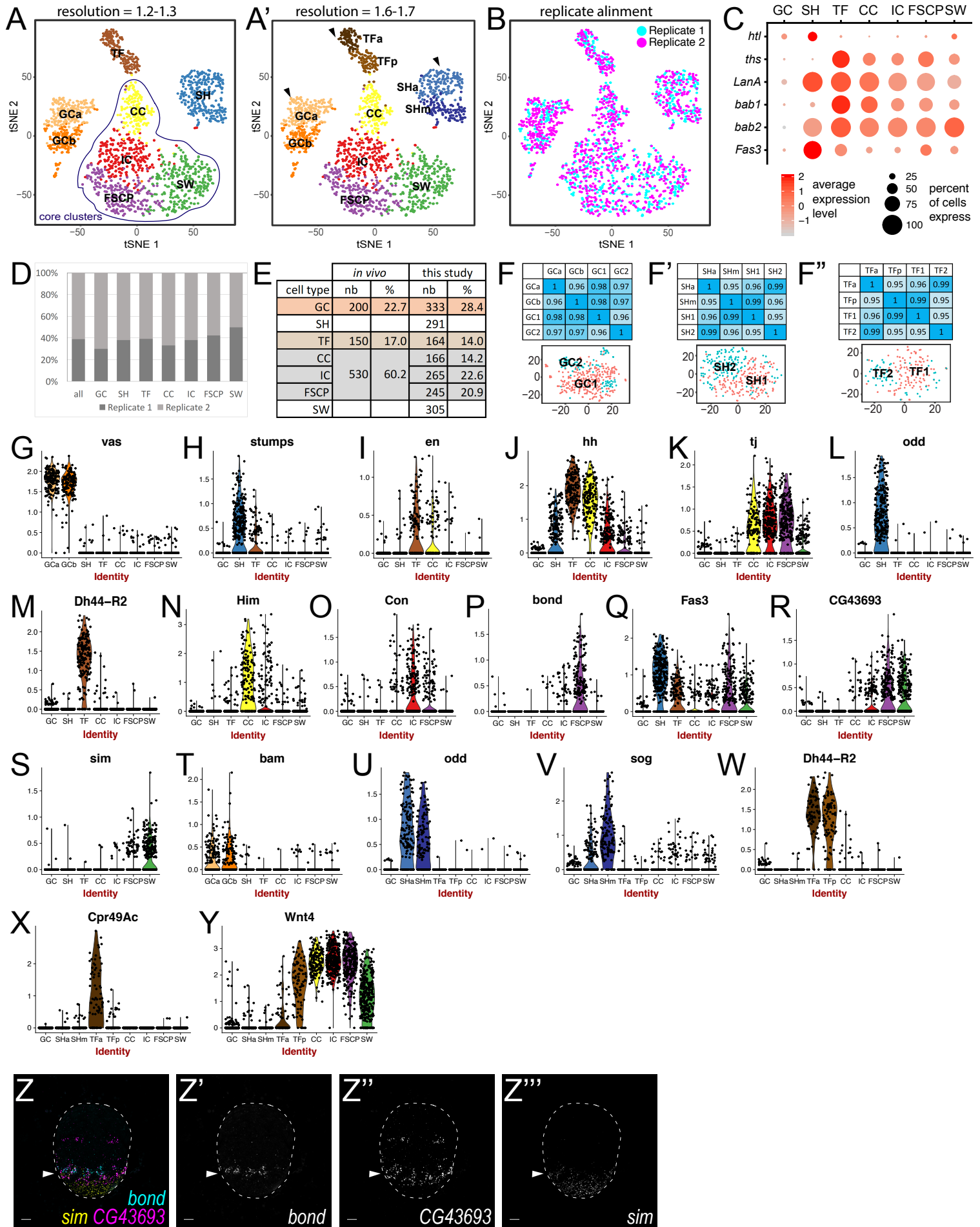

Supplement: Supplemental Material [file supp_gad.330464.119_Supplemental_FigS2.ps]
