# Supplementary material for: A single-cell atlas of the developing Drosophila ovary identifies follicle stem cell progenitors
Source: Genes Dev. 2020 Feb 1;34(3-4):239–49. doi: 10.1101/gad.330464.119 (PMC7000915; doi:10.1101/gad.330464.119)
Supplement: Supplemental Material [file supp_gad.330464.119_Supplemental_FigS3.ps]

Figure S3

A

GC	SH	TF	CC	IC	FSCP	SW	
1.2E-14	0.849	1	1	1	1	1	Ribosome
>1E-14	0.512	0.644	0.603	0.061	0.145	0.094	RNA-binding
>1E-14	0.029	0.062	9.7E-12	1.50E-03	3.6E-10	5.6E-05	Mitochondrial
>1E-14	1	1	0.642	1	1	1	Nuclear-encoded oxidative phosphorylation
9.3E-06	0.161	0.106	0.373	0.276	0.296	0.688	Autophagy-related
>1E-14	0.838	0.937	0.701	0.150	0.072	0.033	Spliceosome
4.94E-10	0.990	0.155	0.355	0.562	0.394	0.718	Ubiquitin-related
4.26E-04	1	0.136	0.964	0.859	0.601	0.710	Phosphatases
3.10E-04	1	1	1	1	1	1	Proteasome
0.167	6.3E-08	9.7E-10	1.8E-04	1.30E-06	1.03E-05	4.89E-05	Major signaling pathways
1	0.003	5.9E-06	0.100	0.260	0.449	0.005	Receptors
1	0.005	2.4E-11	9.2E-09	1.0E-04	3.1E-05	0.173	Trans-membrane proteins
1	9.2E-05	4.5E-08	9.2E-06	0.351	0.697	0.129	Secreted proteins
1.2E-08	1.4E-05	0.168	0.088	2.7E-05	0.005	1.8E-06	Transcription factor/DNA binding
9.6E-07	0.065	6.3E-04	0.180	1	0.745	1.9E-05	Chaperone and heat shock proteins
0.003	2.3E-10	1.3E-12	0.040	0.007	0.024	0.013	Cytoskeletal
1	2.0E-04	1.7E-12	4.3E-11	2.0E-04	3.4E-06	0.023	Glycoproteins
5.8E-04	0.545	2.6E-05	0.002	0.052	0.061	0.062	Kinases

B

	gene	line IDs
SH	*ct*	v 204071
SH	*ct*	bl 27327
SH	*tutl*	bl 66824
SH	*tutl*	bl 39652
SH	*sog*	ky 104415
SH	*CG7860*	ky 103610
SH	*gprk2*	bl 4786
SH	*drm*	bl 7098
SH	*wb*	bl 62797
SH	*wb*	bl 65655
SH	*wb*	bl 62687
SH	*wb*	bl 12665
SH	*cap*	bl 66788
SH	*cap*	ky 105440
SH	*CG32982*	bl 65673
SH	*CG32982*	ky 103659
SH	*nuf*	bl 62802
TF	*hh*	Tanimoto et al.
TF	*en*	bl 30564
TF	*CG17124*	bl 65629
TF	*CG45186*	ky 105238
TF	*CG45186*	ky 113959
TF	*dl*	ky 104671
TF	*dl*	bl 77753
TF	*sick*	ky 103673
TF	*ImpE1*	bl 63324
TF	*glec*	ky 104681
TF	*drak*	ky 105140
TF	*rho*	v 224026
TF	*rho*	bl 26871
TF	*mur89F*	bl 19379
TF	*dh44-R2*	ky 113668
TF	*ppn*	bl 77733
TF	*hh*	bl 67046
TF	*hh*	bl 67493
TF	*CG6231*	bl 76710
TF	*CG10433*	bl 62659
CC	*CG3625*	v 202570
CC	*CG3625*	v 200177
CC	*CG3625*	ky 103616
IC	*con*	v202269
IC	*con*	v202267
IC	*CG3168*	bl 12516
IC	*CG3168*	bl 12731
IC	*CG6414*	v 256582
FSCP	*bond*	bl 65697
FSCP	*CG43693*	ky 105025
FSCP	*kank*	v105146
FSCP	*kank*	bl 65677
FSCP	*kank*	bl 76748
FSCP	*fas3*	ky 104485
FSCP	*heph*	bl 19538
FSCP	*heph*	v 202442
SW	*cv-2*	ky 104532
SW	*sim*	bl 9150
SW	*sim*	v 207019
SW	*obp19d*	bl 66679
SW	*beat-IIIc*	v 203176
SW	*olf413*	bl 77717
SW	*olf413*	v 201719
SW	*trim9*	ky 113356
SW	*trim9*	ky 104724
SW	*trim9*	bl 6682
SW	*nord*	v 202620
SW	*nord*	bl 22671
SW	*hth*	bl 62588
SW	*hth*	bl 65540
SW	*hth*	bl 65547
SW	*hth*	v 205730
SW	*CG32473*	bl 29115
SW	*CG32473*	bl 77725
SW	*mnd*	bl 12791
SW	*mnd*	ky 104442
SW	*rau*	bl 65690
SW	*bnl*	bl 62607
SW	*CG34383*	bl 67473
SW	*kn*	v 203048
SW	*CG4362*	bl 65731
SW	*ds*	bl 67432

cell type specific expression
specific but sparsely expressed
unspecific/broadly expressed
no expression

C

				Tissues with expression						
	driver line	stage	n	SH	TF	CC	IC/EC	FSCP/FC	SW	other tissues
SH	*cut-Gal4*	L3 current	30 (30)	30	5				4	
	v204071	adult-lineage	17 (120)	13	5		1			
TF	*hh-Gal4*	L3 current	15 (15)	30	5				4	
	Tabata lab	adult-lineage	65 (120)		63	13	9	10		
CC	*CG3625-Gal4*	L3 current	18 (18)		7	15	12		14	
	v202570	adult-lineage	34 (120)	4	6	16	4	1		
IC	*con-Gal4*	L3 current	18 (18)		7	2	18	3	8	
	v202269	adult-lineage	65 (150)	2	1	25	52	32		
FSCP	*bond-Gal4*	L3 current	17 (17)				11	16	12	
	bl65697	adult-lineage	32 (100)	5			20	31		
SW	*sim-Gal4*	L3 current	25 (25)		4		7	19	25	
	bl9150	adult lineage	37 (200)	9		1	12	28		peritoneal sheath
